# Genome-wide data from the Bubi of Bioko Island clarifies the Atlantic fringe of the Bantu dispersal

**DOI:** 10.1186/s12864-019-5529-0

**Published:** 2019-03-06

**Authors:** Pere Gelabert, Manuel Ferrando-Bernal, Toni de-Dios, Benedetta Mattorre, Elena Campoy, Amaya Gorostiza, Etienne Patin, Antonio González-Martín, Carles Lalueza-Fox

**Affiliations:** 1 Institute of Evolutionary Biology (CSIC-Universitat Pompeu Fabra), Barcelona, Spain; 2 Laboratory of Anthropology, Department of Biology, University of Florence, Florence, Italy; 3 Department of Biodiversity, Ecology and Evolution, Complutense University of Madrid, Madrid, Spain; 4 Forensic Genetics Laboratory, GENOMICA S.A.U., Pharma Mar Group, Madrid, Spain; 5 Unit of Human Evolutionary Genetics, Department of Genomics & Genetics, Institut Pasteur, Paris, France; 6 CNRS UMR 2000, Paris, France; 7 Center of Bioinformatics, Biostatistics and Integrative Biology, Institut Pasteur, Paris, France

**Keywords:** Bubi, Bioko Island, Bantu-speaking groups, Population genetics, Isolation

## Abstract

**Background:**

Bioko is one of the few islands that exist around Africa, the most genetically diverse continent on the planet. The native Bantu-speaking inhabitants of Bioko, the Bubi, are believed to have colonized the island about 2000 years ago. Here, we sequenced the genome of thirteen Bubi individuals at high coverage and analysed their sequences in comparison to mainland populations from the Gulf of Guinea.

**Results:**

We found that, genetically, the closest mainland population to the Bubi are Bantu-speaking groups from Angola instead the geographically closer groups from Cameroon. The Bubi possess a lower proportion of rainforest hunter-gatherer (RHG) ancestry than most other Bantu-speaking groups. However, their RHG component most likely came from the same source and could have reached them by gene flow from the mainland after island settlement. By studying identity by descent (IBD) genomic blocks and runs of homozygosity (ROHs), we found evidence for a significant level of genetic isolation among the Bubi, isolation that can be attributed to the island effect. Additionally, as this population is known to have one of the highest malaria incidence rates in the world we analysed their genome for malaria-resistant alleles. However, we were unable to detect any specific selective sweeps related to this disease.

**Conclusions:**

By describing their dispersal to the Atlantic islands, the genomic characterization of the Bubi contributes to the understanding of the margins of the massive Bantu migration that shaped all Sub-Saharan African populations.

**Electronic supplementary material:**

The online version of this article (10.1186/s12864-019-5529-0) contains supplementary material, which is available to authorized users.

## Background

**Fig. 1:**
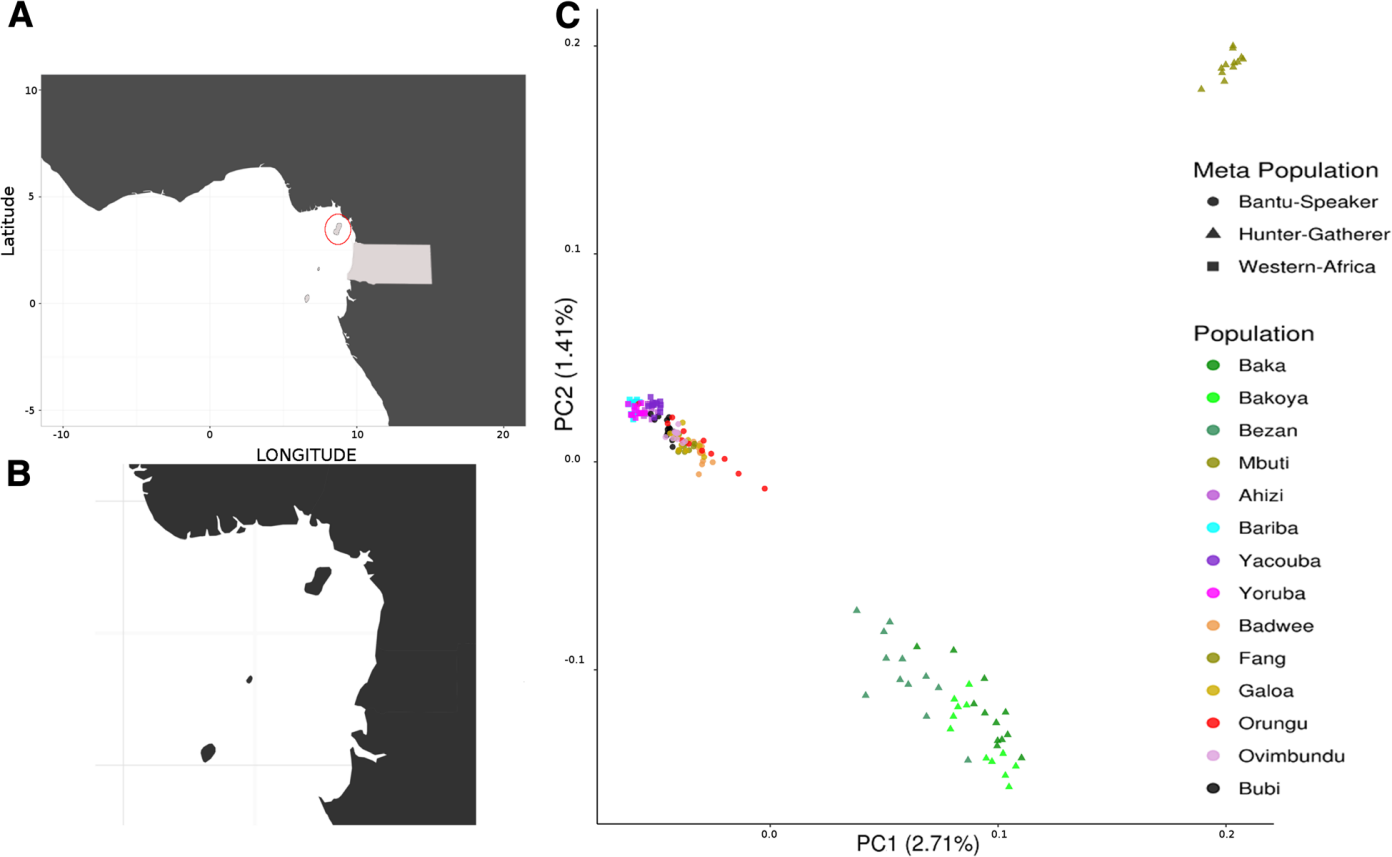
**a** Map of the Gulf of Guinea showing the location of Bioko Island and the neighbouring countries. **b** Map of the Bioko Island. **c** Genotype-based principal components analysis (PCA) plot obtained with EIGENSOFT smartpca. A dataset of 168 individuals from 14 populations and 581,224 SNPs are included. The percentage of variance explained is written along the axes. The maps have been created with R package

The Gulf of Guinea, which covers a large portion of the African coast, is characterized by complex and rich ecosystems. Of the few islands located in Atlantic Africa, four of them are found within the Gulf of Guinea (Fig. 1a). Bioko is the largest of these islands, with a total area of 2017 km^2^ (Fig. 1b). It is located 32 km offshore of Cameroon but constitutes in fact, the northernmost part of Equatorial Guinea, a former Spanish colony. The island is volcanic and very mountainous, with an abrupt coastline; despite its small size, its highest peak has an altitude of 3012 m. The indigenous population of Bioko, the Bubi, speak Bubi, a basal Bantu language [1]. The closest Bantu language on the mainland is most likely Galoa, which is spoken by Bantu-speaking groups of the Ogowe basin in Gabon [2]. The Bubi have a distinct and unique culture among Bantu-speaking people [3], including the belief that different spiritual beings reside in specific geographical locations along the island and the existence of well-defined matrilineal clans [4].

The origin of the Bubi people is controversial. Since the British explorer Richard Francis Burton visited the island (then called Fernando Poo) in 1874 [5], ethnographers generally considered the Bubi to be the original settlers of Bioko. However, it is currently accepted that the Bubi agriculturalists arrived from the mainland in dugout canoes about 2000 years ago during the Late Neolithic [6–8]. Ever since, the Bubi seem to have been isolated from mainland Bantu-speaking groups [9]. Bubi mythology explains that, upon their arrival to the island, they found other, more robust people living there, a population whom they called Balettérimo [1, 9].

In fact, some unsystematic archaeological prospects carried out by Spanish scholars have uncovered pre-Neolithic lithic tools of a typology that has been called banapense, although this lithic typology does not currently have a clear chronological framework [9].

The expansion of the Bantu-speaking farming communities is probably one of the most important human movements that have taken place in recent African history [10]. This movement started approximately 4000 to 5000 years ago [11], likely from a source close to the present-day North Cameroonian [12] or Gabonese/Angolan Bantu-speaking populations [13], depending on which model – “early-split” or “late-split” – is assumed. While the first model suggests that the Bantus made an early separation into western and eastern branches, the second model supports an initial movement south across the rainforest before splitting into two branches, one headed south and the other east. Whatever the route of dispersal, the Bantu-speaking migration triggered an expansion of agriculture and ironwork along with the spread of Bantu languages (part of the Niger-Congo family) across most of Central, South and East Africa [13–16].

During their extensive geographical migration, the Bantus encountered and admixed with local rainforest hunter-gatherer (RHG) tribes. Several Bantu-speaking groups have been studied from a genetic point of view over the years, especially using mitochondrial DNA (mtDNA) and Y chromosome markers [12, 17–20]. These studies have detected a substantial fraction of Pygmy mtDNA lineages within the Bantu speakers, but rarely the opposite [17, 18]. For example, traces (around 1%) of RHG Y chromosomes have been found in Bantu-speaking groups from Gabon and Cameroon [12] and signals of hunter-gatherer Khoisan Y chromosomes in Bantu-speaking groups from Mozambique [21].

Despite these efforts, the genetic patterns of variation within the Bantus remained largely unexplored on a genomic scale until fairly recently [14, 15, 22]. The analysis of a large and geographically diverse dataset of 35 Bantu groups has recently confirmed the existence of this RHG ancestry as well as the acquisition of adaptive alleles from these local populations, especially at the human-leukocyte antigen (HLA) loci. By measuring the length of the introgressed genetic fragments, the admixture between western Bantu-speaking populations and RHG was estimated to occur about 800 years ago, mostly after Bantu-speaking populations began moving throughout Sub-Saharan Africa [13]. According to this study, while the most likely parental source population of Bantu ancestry in both eastern and southern Bantus was located in northern Angola, Bakoya of Gabon and Congo were the best parental source for RHG ancestry. Recent retrieval of ancient genomes from different African localities, notably in the south and the east, could help elucidate these past admixture events [23, 24]. So far, however, no ancient genomes have been retrieved from the Gulf of Guinea.

There are hypothesis that could potentially be explored with genome-wide data from the Bubi. First, their relatively long period of isolation on the island is a potential way to test the age and extent of the Bantu admixture with RHG tribes, an event that supposedly took place among the coastal Bantu groups after isolation of Bubi ancestors on the island. Second, studying a tribe from one of the few islands around Africa could provide information about potential effects of endogamy and isolation that are less likely to occur in mainland tribes. Finally, genome-wide data from the Bubi can offer clues regarding the adaptation of this group to local conditions. For example, it is known that the population of Equatorial Guinea has one of the highest levels of malaria infection in the world [25] and ranks 13th in the list of malaria prevalence countries, representing the second highest cause of death in the country [26] Despite prevention efforts carried out since 2004, severe malaria prevalence in Bioko children remains high [27]. Thus, screening potential resistant variants can help us further understand the selective pressures faced by the Bubi during the last few hundred years.

We show in this work that the Bantu population of Bioko Island mirrors the genetic makeup of the extant, coastal Bantu-speaking groups, also clarifying the dynamics of this human expansion into the Atlantic islands of Africa.

## Results

We sequenced 13 Bubi genomes obtaining a depth of coverage up to 21x-32x (Additional file 1: Table S5). All mtDNA haplogroups present in the Bubi are subclades of the L haplogroup (L1b, L2b, L3e, L3f, and L3e) and are common in other populations from the Gulf of Guinea. All male individuals of this dataset belong to subclades of the E1b1a1 haplogroup, the predominant Y-chromosome lineage in Western, Central and Southern Africa (Additional file 1: Table S6).

On a genome-wide scale, the first two components of the principal component analysis (PCA) separate the RHG from the Bantu speakers and the Western Africa non-Bantu populations (Fig. 1c). When PC3 and PC4 are plotted, Bantu-speaking and Western African populations cluster separately (Additional file 2: Figure S1). Three Bubi individuals that we named Bubi-subset1 (BBS014, BBS018, BBS023) fall within the Western Africa cluster showing that some individuals share a larger proportion of Western-Africa ancestry than others, while the rest (named Bubi-subset2) cluster within the Bantu diversity (Additional file 2: Figure S1). To examine if this clustering reveals the presence of population substructure, we have used the USCS liftover [28] to convert the chimpanzee reference sequence (*Pan troglodytes* 3.0 assembly, GCA_000001515.5) to human coordinates in 546,558 single nucleotide polymorphisms (SNPs) of our dataset. We have subsequently computed f_4_ statistics in the form (Bubi-subset1, Bubi-subset2; *X*, chimpanzee), to examine the homogeneity of the Bubi population (Additional file 2: Figure S2, Additional file 1: Table S7). None of the tested populations showed elevated (| > 3|) values of Z-score; therefore we have treated the Bubi as a single population in subsequent analyses.

**Fig. 2:**
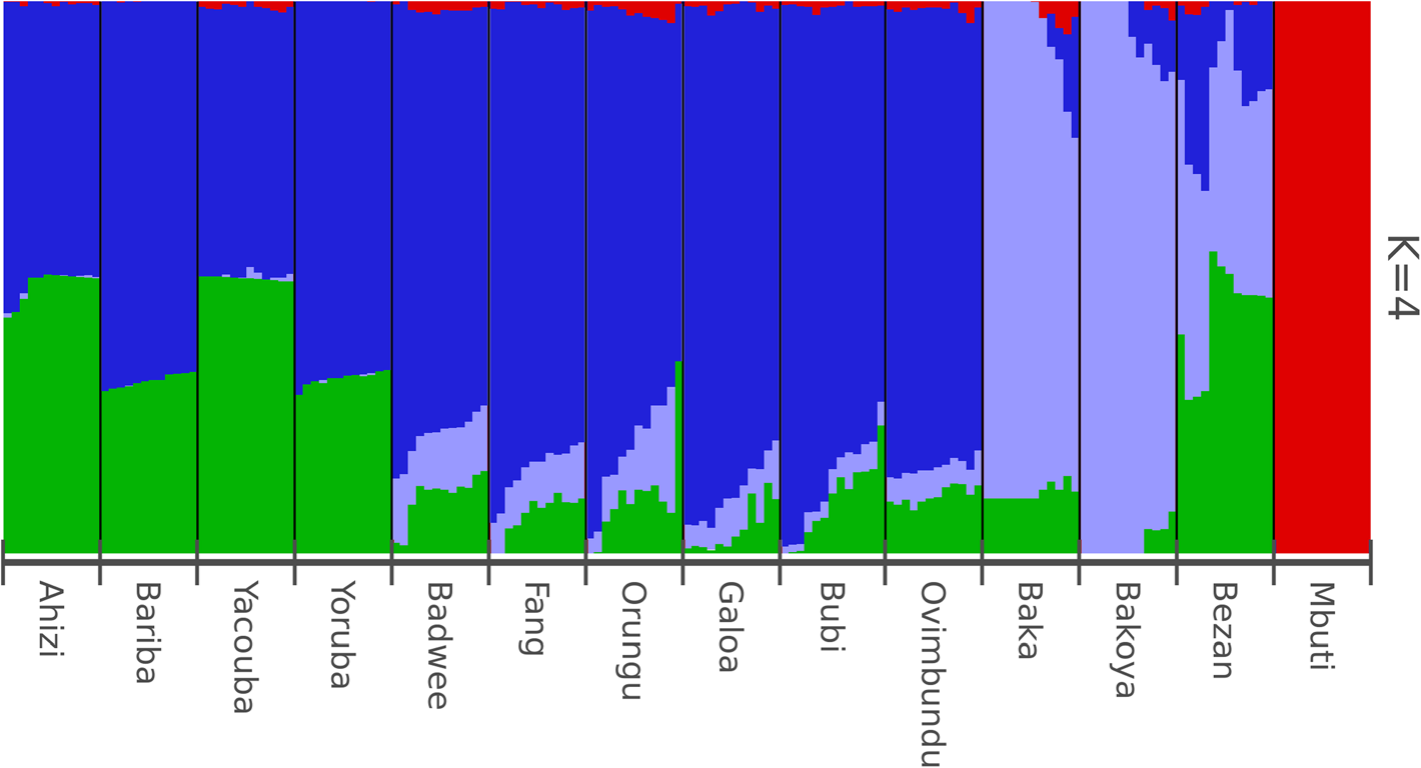
Graphical representation of shared genetic components performed using the ADMIXTURE (K = 4) software: 169 individuals from 13 populations and 456,095 SNPs have been plotted. Although the cross-validation errors show the lowest value at K = 2, we have chosen K = 4 because it is the first plot where the crucial RHG, Bantu and Western African components are clearly identified

Moreover, ADMIXTURE analysis (K = 4) (Fig. 2 and Additional file 2: Figure S3) shows that the Bubi contain the same components as the other Bantu-speaking populations of the dataset but lower levels of the RHG component (in grey) than most of the mainland Bantu-speaking populations (with those from Angola being the exception). Some of the remaining ADMIXTURE plots (K = 2–15) (Additional file 2: Figure S4) also indicate potential substructuring among the Bubi; however, the sample size is too small to test if this is correlated with geography.

**Fig. 3:**
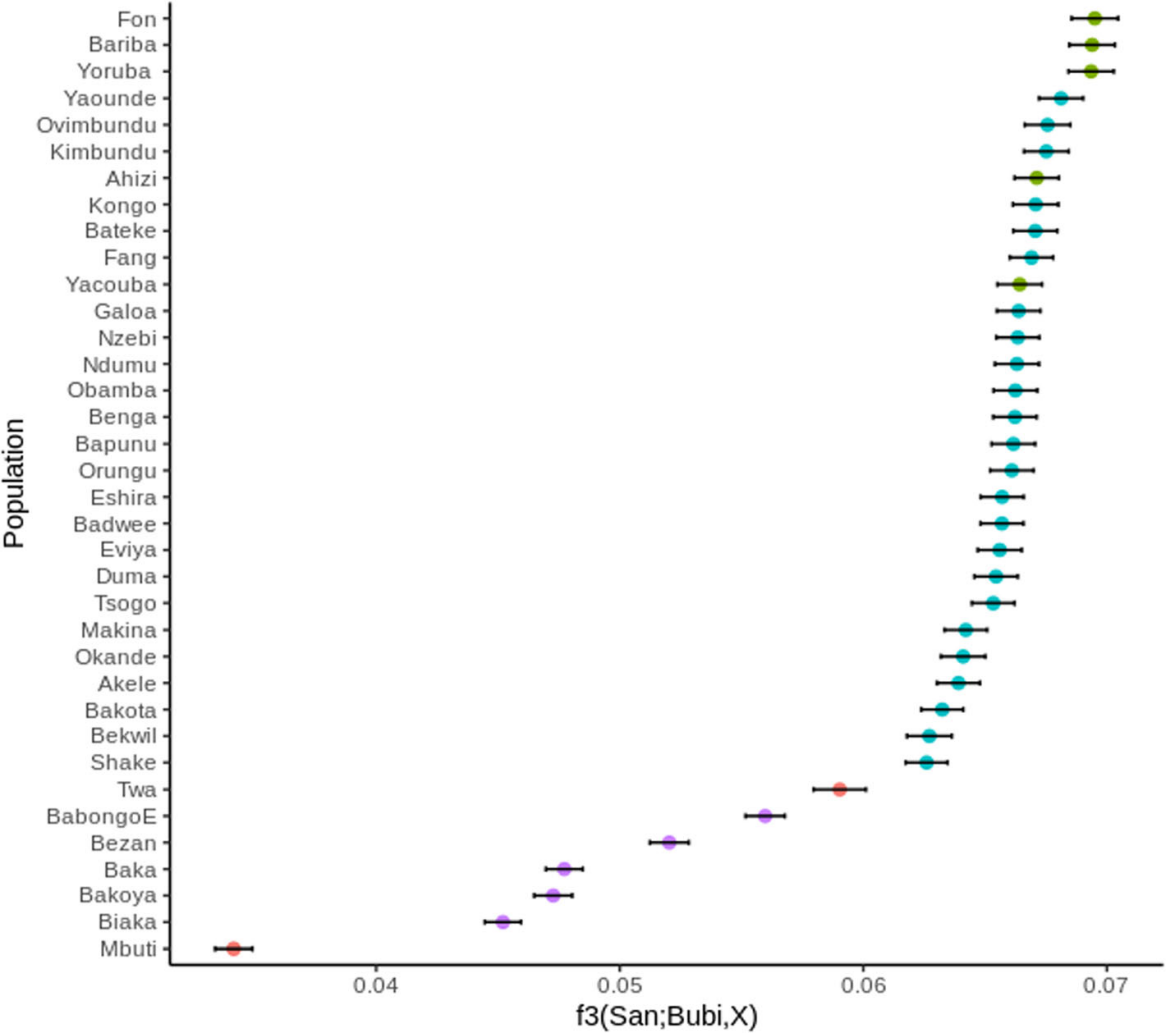
Statistic f_3_values obtained with popstats when taking San as outgroup. The statistic and one standard error deviation are presented for each combination test. Blue dots represent Bantu-speaking populations, Green dots represent Western-African populations, orange dots Represent Eastern Hunter-Gatherer populations and purple dots indicate Western Hunter-gatherer populations

To determine which mainland populations share a higher level of genetic ancestry with the Bubi, we tested all neighbouring populations with the outgroup f_3_ statistic (considering San as outgroup). Our results indicate that non-Bantu Western African populations such as Yoruba, Bariba, Fon and Azhizi, as well as Bantu-speaking groups from Angola such as Kongo, Ovimbundu and Kimbundu show more genetic affinities with the Bubi, apparently because they all show lower hunter-gatherer ancestry as compared to other groups (Fig. 3 and Additional file 1: Table S8). To explore the possible source of genetic admixture in the Bubi, we have also calculated the f_4_ statistic for the combinations (*Test*, San; Bubi, Mbuti), (*Test*, San; Bubi, Baka), (*Test*, San; Bubi, Yoruba), (*Test*, San; Bubi, Fang) (Additional file 2: Figure S5A-D)*.* This selection represents the four major genomic components in the Gulf of Guinea. We have found that the Bubi show the highest levels of shared ancestry with the Western African populations, which do not overlap with other populations when the comparison is established with RHG components. Angolan Bantu-speaking populations such as Ovimbundu, Kongo and Kimbundu also show elevated levels of shared genetic ancestry (Additional file 2: Figure S5). Bubi people seem to have very low levels of genetic admixture from the RHG populations; however, this small signal is absent in Western Africans.

Furthermore, owing to the colonial history of Bioko we have explored the possibility of some Iberian contribution to the Bubi ancestry by calculating the f_3_ statistic in the form (Iberia, *X*; Mbuti). The Bubi are placed within the range of Western African and other Bantu-speaking populations (Additional file 2: Figure S6); therefore, no Iberian genetic affinities can be discerned within the current dataset.

**Fig. 4:**
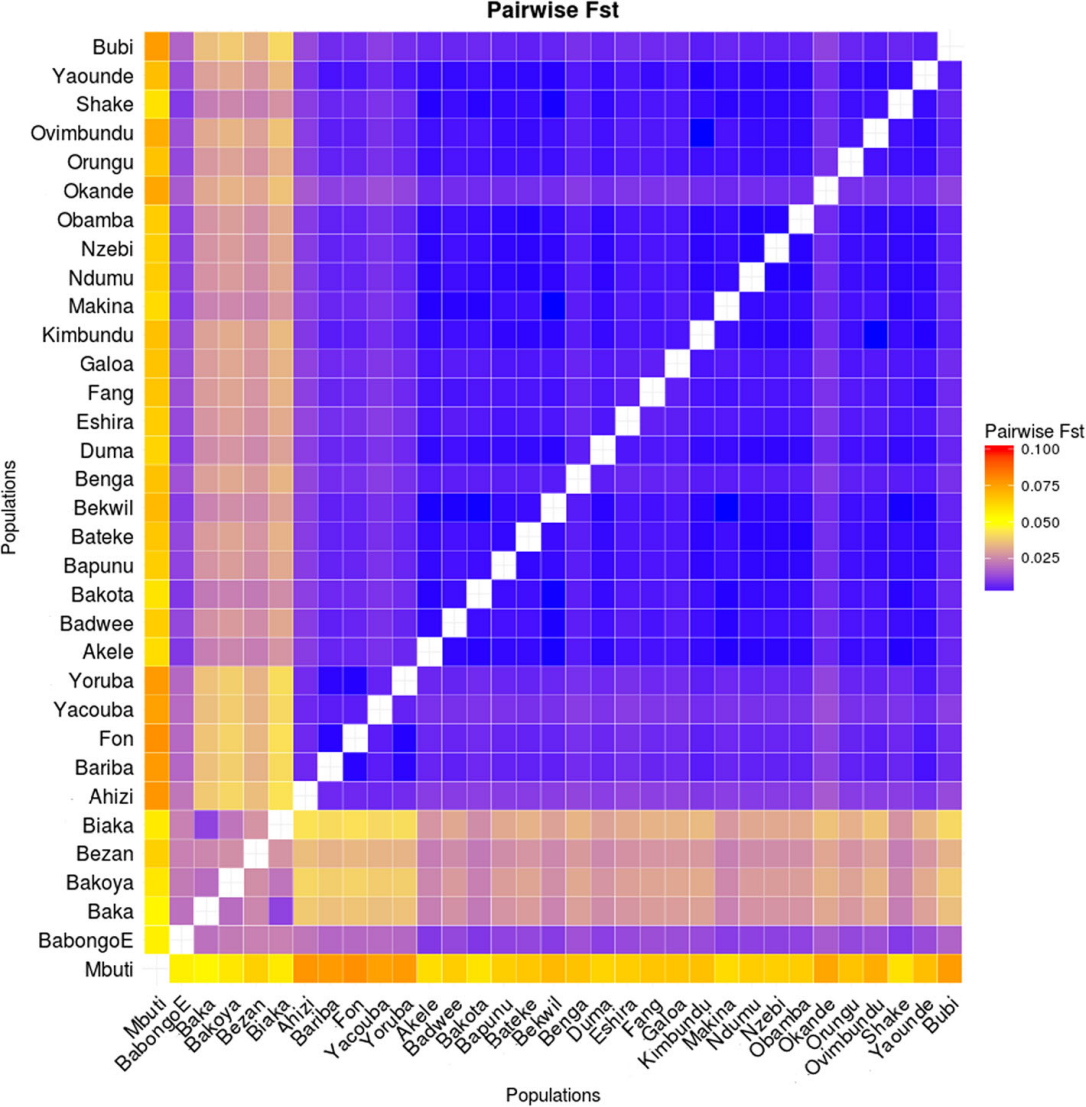
Matrix of pairwise F_st_ calculations. The F_st_ values have been calculated using plink and with a dataset of 592,395 SNPs

Pairwise F_st_ is a statistic used to measure population differentiation based on allelic frequencies. We used this test to quantify the level of genetic differentiation between all combinations of populations in our dataset. Low levels of F_st_ statistic indicate that the tested populations share a large proportion of the genotypes, while high levels are indicators of genetic differentiation. Among all African samples, RHG tribes appear to have the highest levels of genetic differentiation, both among themselves and with the agriculturalist groups (Fig. 4). The lowest values of genome-wide F_st_ for the Bubi are again with Bantu-speaking Angolan groups (Kimbundu, F_st_ = 0.0045; Ovimbundu, F_st_ = 0.0045), and also with a Bantu-speaking group from Cameroon (Yaounde, F_st_ = 0.0045). On the other hand, we found that the Bubi displayed the highest F_st_ values when compared with RHG populations (Additional file 1: Table S9).

**Fig. 5:**
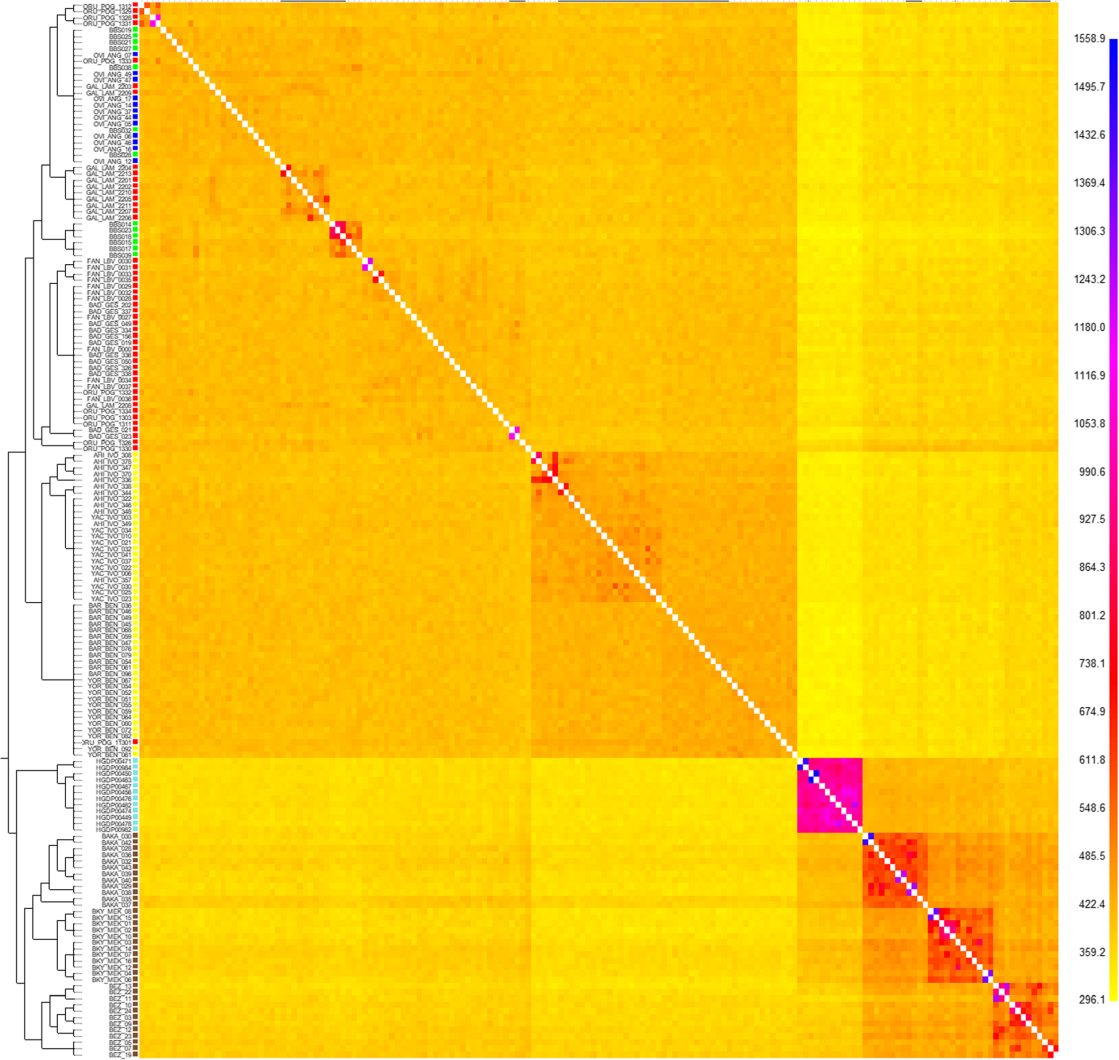
Matrix of shared counts of haplotypes obtained using fineSTRUCTURE. The dataset includes 491,203 SNPs and 169 individuals. The structure of the matrix has been adapted using the fineSTRUCTURE dendrogram. Green label represents the Bubi individuals, red label is used to represent the Gabon-Cameroon Bantu-speaking individuals, Blue label represents the Angolan Bantu-speaking individuals, Yellow label represents the Western-African individuals, Light blue label represents the Eastern Hunter-Gatherer individuals and Brown label represents the and Western Hunter-gatherer populations

FineSTRUCTURE uses the coancestry matrix obtained from ChromoPainter to classify samples based on haplotype diversity. Using this approach, the matrix and the resulting dendrogram confirm that the closest populations to the Bubi are the Ovimbundu (Fig. 5 and Additional file 2: Figure S7), but also some Gabonese and Cameroonian Bantu-speaking populations. Interestingly, the Bubi are divided into two different clusters (Additional file 1: Table S7), one including only individuals from Bioko Island and the other shared with other Bantu-speaking groups. This, again, suggests some level of substructure in the population. Interestingly, the RHG show a higher level of haplotype differentiation compared to other populations. To additionally test for the presence of Iberian ancestry we have repeated the fineSTRUCTURE analysis including 12 Iberian individuals (Additionlal file 2: Fig. S8), following the same methodology previously described and 244,897 phased SNPs.

To explore possible admixture events that could have led to the origins of the Bubi, we also performed GLOBETROTTER analysis (based on ChromoPainter); however, no admixing events could be identified. We also used fineSTRUCTURE to plot a PCA based on haplotype differentiation. This method clusters the populations into the same groups as those used when considering genotypes. In this analysis, the Bubi present a clear intermediate position between Western African and Bantu-speaking populations (Additional file 2: Figure S9). For computational convenience, we performed these analyses with a restricted dataset.

**Fig. 6:**
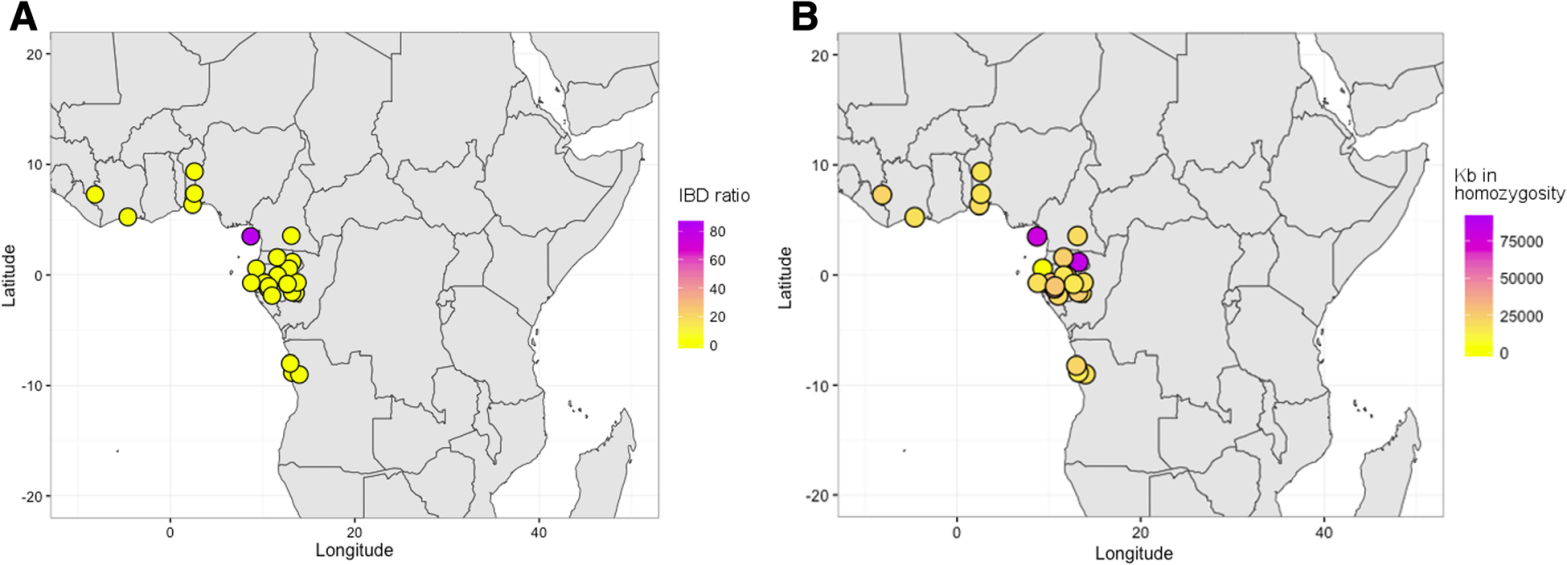
**a** Average of the genome in homozygosity (in kb) for the Bubi and mainland Bantu populations. **b** Average of the shared IBD genomic blocks between the Bubi and mainland Bantu populations (IBDs > 2 cM). The maps have been created with R package

Furthermore, we also estimated identity by descent (IBD) tracks to help elucidate the recent co-ancestry links between the Bubi and mainland populations. We found the Bubi to share the highest number of IBD blocks longer than 2 cM (indicative of genealogical connections occurring during the last 2500 years [29]) with populations from Angola (Ovimbundu, Kongo), as well as some from Gabon (e.g., Obamba, Duma, Bateke and Bapunu) (Fig. 6 and Additional file 1: Table S10). Additionally, we estimated the average of runs of homozygosity (ROHs) in each population, as well as the fraction of the genome in homozygosity as signals of endogamy. We found that the Bubi are the second most endogamous Bantu-speaking group (Additional file 1: Table S11) after the Bekwil population, although the ROHs of RHG tribes were longer on average than those observed among the agriculturalist groups (Additional file 2: Figure S10, Additional file 1: Table S12).

We found that all Bubi individuals possessed the malaria-resistant allele of the *ACKR1* [30] and *CD36* genes [31], in addition to certain variations in other genes such as *G6PD* [32], *ATP2B4* [33], *GRK5* [34]*,* and *IL-10* [35]. Moreover, resistant variants are absent in *ABO*, *HBB* [36] and *TIRAP* [37] (Additional file 1: Table S13) [38]. However, considering these mutations are observed at low frequency among other African groups, it is likely they were simply not present in the ancestors of the Bubi (Additional file 1: Table S14). We compared the allelic frequencies of malaria SNPs in the Bubi with those from neighbouring populations of the Gulf of Guinea – such as Esan, Gambian, Mende, and Yoruba – for which genome-wide sequence data was available. We found statistically significant (Fisher’s exact test) differences in some alleles, but nothing that indicated a unique trend in the Bubi (Additional file 1: Table S15). We subsequently conducted a genome-wide F_st_ scan between the Bubi and Yoruba, using all the variable positions with MAF > 0.05 and missing genotypes < 0.05, plotting the mean F_st_ values in 0.5 Mb windows. We set a threshold of significance in 0.25 [39]. We have failed to detect any signal of a selective event, including those regions related to immunity against malaria (Additional file 2: Figure S11).

## Discussion

Our genomic study of the population of Bioko Island confirms that the Bantu-speaking migration that shaped most of the present-day human diversity in Sub-Saharan Africa [40] also had a significant impact on African islands of the Gulf of Guinea. The general components of ancestry found in the Bubi are not different from those found in mainland Bantu-speaking groups, although in the case of the Bubi, the RHG ancestry is lower than the amount detected in most Western Bantu-speaking groups. Moreover, we did not detect a significant difference in the origin of the Bubi RHG genetic signal to the one observed in other Bantu populations. One potential explanation could be that an admixture event between the ancestors of the Bubi and the RHG tribes started about 2000 years ago and was brought to the island upon settlement, but continued to increase thereafter in most mainland Bantu-speaking groups. It is worth noting that the time of admixture can be underestimated when using methods based on linkage-disequilibrium decay if continuous admixture events actually occurred [41]. Therefore, the current 800 years estimate [13] could in fact be the end of a long period of gene flow between mainland Bantu-speakers and RHG. This scenario could help explain the clustering of the Bubi with Western African groups in some analyses (the latter groups also show residual or no traces of RHG ancestry).

Due to a certain degree of heterogeneity detected within the Bubi that was evident from PCA, ADMIXTURE and fineSTRUCTURE analyses, the possibility that different populations from Bioko could harbour slightly different genetic histories existed. Notably, some Bubi show almost no signs of RHG; interestingly, one of these individuals is from Bariobé, a relatively isolated province in the mountainous interior of the island. An alternative possibility could be that the small fraction of RHG ancestry was acquired by gene flow from coastal regions after the ancestors of the Bubi settled in Bioko. For example, the presence of both Fang and Benga people in Bioko has been described in historical times, partly related to the slave trade. In fact, although the slave trade was not so important in Bioko, it was very active in other coastal centres of the Gulf of Guinea, especially in some of the minor islands such as Corisco and Annobón [42]. Nonetheless, due to the cultural particularities of the Bubi and the clear genetic signals of endogamy and isolation, it seems unlikely they would assimilate a significant number of foreign people. In addition, no signals of potential Iberian admixture have been detected among the Bubi.

Within the Bantu-speakers, the Bubi are more closely related to Angolan than to the geographically closer Cameroon groups (this is supported for instance by fineSTRUCTURE or f_3_ statistics). Based on the evidence that Bantu expansion likely moved from Angola northwards [13], it is possible that Bantu-speaking groups from Cameroon experienced subsequent admixture events with neighbouring RHG populations.

The Bubi particularities are mirrored by their geographically induced genetic isolation as well as their linguistic differences with neighbouring, mainland populations. At the linguistic level, the Bubi language is basal to most Bantu languages [40, 43] and clusters together with northwest Bantu speakers. This correlates with archaeological findings from the region dated from 5000 to 2500 years ago and associated to the spread of Bantu languages [40]. This decoupling between language and genetics could be explained if the former was acquired by or imposed onto the Bubi mainland ancestors. Accordingly, there are some historical accounts that consider the Bubi to be an enslaved tribe that escaped to Bioko [44].

The Bubi seem to have experienced a certain history of isolation that left a mark in their genomes. Out of all the Bantu-speaking groups, for instance, we found that the Bubi have some of the highest levels of IBD tracks shared among members of the same population, a signal of low diversity that is compatible with endogamy. In fact, in the ROH analysis, the Bubi rank as the second most endogamous Bantu-speaking group, only after the Bekwil. Nevertheless, the fact that the Bubi do not show a large genetic differentiation from potential source populations along the coast also indicates that drift did not have time to operate at large scale and that colonization of the island did not occur a long time ago.

The Bubi, like other groups from the Gulf of Guinea, display a high frequency of some mutations associated with protection against malaria. Other mutations, however, are absent or segregating. The underlying mutation for the Duffy-negative phenotype (at the *ACKR1* gene) that is known to protect against *Plasmodium vivax* and *P. knowlesi*, seems to be fixed, or at least is present at extremely high frequencies, in the Bubi population. In fact, this is a common trait in all Western Africa. At the beginning of the twenty-first century, malaria was responsible for a child mortality rate of 152 per every 1000 births in Bioko island, a figure that is only beginning to decrease thanks to recent malaria control projects [27]. However, in a genome-wide scan performed against the Yoruba, we were unable to identify genomic regions in the Bubi that appear to be shaped by natural selection, even despite their insular conditions.

However, due to the limited sampling size and restricted distribution within Bioko, our study has to be considered as a preliminary assessment of the current Bubi genetic diversity. Despite evidences that our sampling size can effectively estimate parameters of genetic diversity from a larger population (see Methods last section), additional sampling with a broader geographical distribution should be undertaken in the future.

## Conclusions

In addition to the general population affinities of the Bubi, we have unveiled genetic evidences of a certain degree of isolation, which can be related to the insular conditions; this trait is quite unique in most of African mainland populations. Our study of the genomic composition of the Bubi not only adds further information to the current genetic diversity within Africa and its Atlantic islands, but also points to the importance of the genome-wide analyses in unravelling population affinities, selective pressures and past migrations that can be correlated with linguistic and archaeological information. We conclude that the origins of the Bantu expansion still needs further research and that future retrieval of ancient genomes from Central and Western Africa could shed need light on the cradle of the Bantu migrations.

## Methods

### Samples

All thirteen individuals analysed in this study are members of the Cultural Bubi Association of Fuenlabrada, Madrid (Spain). We obtained informed consent from all subjects. We discarded 25 of the interviewed individuals because of admixed ancestry; many of them had a recent Fang ancestor from the mainland. Even though most of the individuals were not born in Bioko, we verified that the selected individuals had all grandparents born in the island; many of the volunteers’ direct ancestors come from Malabo, Bariobé and Baney, which are located in the Northeast region of Bioko (Additional file 1: Table S1).

### Extraction, sequencing and mapping

We isolated DNA from cotton swabs using all the available material and an organic-based DNA extraction method adapted to Amicon® Ultra 0.5-mL columns [45]. After extraction, we concentrated the DNA by centrifugation up to 50 μL and subjected samples to a quality control. To ensure there was a proper DNA concentration, 1 μL of sample was loaded in a 1% agarose gel and stained with ethidium bromide. Only a single band was observed. The samples were quantified with BioTek’s Epoch and yielded values, on average, of 68.88 ng/μL.

Genomic DNA libraries were prepared using TruSeq DNA PCR-Free Library Preparation Kit (in accordance with the general settings of the preparation guide). The procedure produced a PCR-free library with 350 bp average insert size that requires 20 ng/ul (in 50 ul samples). DNA samples were randomly fragmented by Covaris system and sequenced in HiSeqX10 (Illumina) with hiseq2x150bp settings plus 65 bp paired-end adapters at Macrogen (South Korea).

We evaluated the paired-end sequenced reads with FASTQc to check their quality. The sequencing adapters were then removed using Adapter removal [46], reads shorter than 30 bp were removed, and the reads were mapped against the Human reference genome [National Center for Biotechnology Information (NCBI) 37, hg19] using Burrows-Wheeler Aligner (BWA) with default parameters [47]. Duplicated reads were removed using Picard Tools MarkDuplicates version 2.8.3 and low quality mapping reads (< 30) were removed with SAMtools version 1.623 [48].

### Genotyping and quality control tests

Unique aligned reads were processed with Base Quality Score Recalibration (BQSR) implemented in the GATK version 3.7 software [49]. Even if the plots did not show signals of systematic errors, we applied recalibration to all filtered reads. We used GATK HaplotypeCaller in GVCF mode for scalable variant calling (using the GRCh37 as a reference sequence). Individual variant calls were merged in a single VCF file using GATK genotypeGVCFs tool, and the variants were filtered using Variant Quality Score Recalibration (VQSR) with a filter level of 99%. We used QD, MQ ReadPosRankSum, FS, and SOR annotations in this step. We excluded any variant with less than 70% of the main depth coverage or more than 200%. We also removed those variants exhibiting qualities below 30. We removed those called variants with a minimum allele frequency below 0.05 and a of Hardy-Weinberg disequilibrium *p*-value below 1e-6.

### Population genetics dataset

We merged our filtered variants with 690,739 SNPs from 1235 genotyped individuals belonging to 35 Western Africa populations. This dataset includes: Bantu-speaking populations, hunter-gatherers and Western African groups [13],using Plink 1.9 [50] (Additional file 1: Table S2). We excluded triallelic sites, A/T and C/G mutations and all sites with a minor allele frequency (MAF) below 0.05. We subsequently removed positions with > 10% missing data and those individuals with > 5% missing values. To ensure that genotypes were properly called after merging the dataset, the Yoruba SNP genotypes were compared against the 1000 Genomes Yoruba population. However, subsequent analyses were performed only with the Yoruba genotypes from Western Africa dataset [51]. Positions that exhibited > 0.2 values of pairwise F_st_ between both samples were also removed. Based on the colonial history of Bioko, we have assessed the presence of potential genetic admixture of the Bubi with Spanish individuals, adding Iberian samples from 1000 Genomes [52] to the SNP dataset. After this procedure, we again removed positions with MAF below 0.05, missing data above 0.1 and Hardy-Weinberg disequilibrium *p*-values below 1e-10.

For most of the analyses, we have extracted a sub-dataset with representative populations from Western and Central Africa. This reduced dataset includes 14 populations and 169 individuals (Additional file 1: Table S3). Some of the population genomics analysis require an unrelated outgroup to the tested populations. We have merged our genotypes with data of eleven San individuals [53] from the Human Origins array [54], followed with the same merging procedure previously detailed. The resulting African dataset –including the Bubi- comprises 130,647 SNPs present in 1259 individuals.

### Mitochondrial (mt) DNA and Y-chromosome analysis

Reads were mapped against the Revised Cambridge Reference Sequence (rCRS) of the human mtDNA [55]. After calling variants with GATK version 3.7 [49] as has been previously described, the mtDNA haplogroups were predicted using Haplogrep version 2 [56]. Y chromosome haplogroups were predicted by classifying the observed mutations according to the PhyloTree database [57].

### Population genomic analyses

To situate the Bubi within the present diversity of the Gulf of Guinea and Western Africa, a principal components analysis (PCA) with the reduced dataset was generated using EIGENSOFT software [58], Results were plotted using R package ggplot2 [59, 60].

ADMIXTURE plots were generated to estimate the proportions of K ancestral components on each individual genome [61] of the reduced dataset. As the analysis assumes linkage disequilibrium (LD), we pruned the dataset. We used Plink 1.9 to remove SNPs with an LD > r^2^ = 0.5 in windows of 50 SNPs. ADMIXTURE analyses were performed with K from 2 to 15 and were repeated five times. The ADMIXTURE iterations were consolidated using CLUMPP with the large K greedy algorithm [62] and the results were plotted using R package *pophelper* [63].

Outgroup f_3_ statistic is a useful test to determine the closest population to a target one using one outgroup population and measuring the amount of shared genetic drift with a test population. San were selected as outgroup, as they represent the most distant African population with genome-wide data, Bubi population was compared to all other populations in the dataset. The f_3_ (San; Bubi, *Test*) statistic was calculated with popstats [64] and the results were again plotted using R. *f* statistics can also be implemented in order to determine which populations exhibited the highest genetic drift with the Bubi people, to do so, we used the popstats software to compute the f_4_ statistic (*Test,* San; Bubi, Mbuti), (*Test,* San; Bubi, Baka), (*Test,* San; Bubi, Yoruba), (*Test*, San; Bubi, Fang)*.* These combinations allow us to dissect the genetic admixture of the tested populations with the Bubi in relation to all the representative source of genetic ancestry in Western Africa: Eastern RHG, Western RHG, Western-African populations and Bantu-speaking populations.

The fixation index (F_st_) is a measure of population differentiation. We calculated the mean pairwise F_st_ values between all the populations present in the global dataset. All autosomal SNPs were included in this analysis using the approach of Cockerham and Weir integrated in Plink 1.9 [65]

The reduced dataset was phased with SHAPEIT2 [66], using 500 states, 50 MCMC main steps, 10 burn-in and 10 pruning steps; recombination maps were interpolated from the HapMap phase 2 genetic maps. After excluding all positions with at least one missing site, we ended up with a dataset of 491,203 variable positions with no missing data.

We used CHROMOPAINTER to build a coancestry matrix based on haplotype data from the phased-reduced dataset. This software estimates the admixture proportions in recipient chromosomes by painting the proportion of each genetic component from the donor populations. We ran CHROMOPAINTER with linked data, estimating n and M parameters through an observation run with no prefixed parameters and including 30 randomly selected samples and three randomly selected chromosomes; fineSTRUCTURE analysis was performed with the counts obtained in CHROMOPAINTER and ran with 1000,000 Markov chain Monte Carlo (MCMC) iterations and output printed every 10,000 iterations. The best tree was calculated with 10,000 state attempts. We also generated a haplotype-based PCA with fineSTRUCTURE.

To identify any admixture events between Bubi ancestors and other populations during the last 4500 years, we used the GLOBETROTTER [41] software on the basis of the defined clusters from fineSTRUCTURE (Additional file 1: Table S4).

### Identity by descent (IBD) analysis

Identity by descent (IBD) blocks are defined as identical chromosome fragments present in multiple individuals that have been inherited from the same ancestral chromosome [67]. We have used RefinedIBD software [68] setting “ibdcm” = 0.5, “ibdtrim” = 62, “ibdwindow” = 2478, and “overlap” = 413; the rest of the parameters were assigned by default. All IBD blocks longer than five centimorgans (cM) were kept and the statistical threshold marked by LOD (the base 10 log of the likelihood ratio of the IBD segments, which is a figure that will depend of the size of the database and the genetic diversity within it) was assigned by default (> 3). The number of SNPs used here was 685,382. We then filtered the IBD segments to keep only those that were shared by any Bubi and another individual of the dataset (including the IBD fragments shared by two Bubi individuals). To reduce the impact that the population size could have on the global counts of IBD blocks per population, we corrected the value of the shared IBD fragments (IBDn) by the population size (t). In order to obtain the average of the IBD blocks shared by any Bubi with any other individual or population, we divided each number obtained in the previous step by the number of Bubi individuals, 13:ratioBubi_pop = (IBDn/t)/13.

### Runs of homozygosity (ROHs) analysis

ROHs (> 1000 kb) were estimated with Plink software. First, we calculated the average (in kilobases) of the genome that it is in homozygosis for each population. Second, we calculated the average of the number of genomic fragments that are in homozygosis for each population.

### Malaria resistance

Relevant mutations associated with malaria resistance in 10 different genes (Additional file 1: Table S11) – as found in genome-wide association studies (GWAS) and other previous studies [31, 69] – were genotyped in Bubi and the 1000 Genomes African populations. Fisher’s exact test was used to determine the statistical significance of the observed differences (*p* < 0.001).

### Evaluation of the effects of limited sample size

We have used a whole genome F_st_ approach to evaluate the effects of the small sample size used in this work. We have randomly grouped the 186 Yoruba individuals from 1000 Genomes in 14 subsamples of 13–17 individuals and wehave estimated the mean pairwise F_st_ values among all population combinations. All autosomal SNPs were included in this analysis using the approach of Cockerham and Weir integrated in Plink 1.9 [65]. No comparison has shown values of mean pairwise F_st_ higher than 0.1, which indicates that the sub samples do not show significant differences in terms of genetic diversity (Additional file 2: Figure S12). This result suggests that the limited Bubi sample size can be used to infer genetic diversity at a higher population level.

## Additional files

Additional file 1: Tables. (XLSX 78 kb)

Additional file 2: Supplementary figures. (PDF 1206 kb)

## Abbreviations

BQSR: Base Quality Score Recalibration
Fst: Fixation index
GWAS: Genome-wide association study
HLA: Human-leukocyte antigen
IBD: Identity by descent
LD: Linkage disequilibrium
MAF: Minor allele frequency
MCMC: Markov chain Monte Carlo
mtDNA: Mitochondrial DNA
NCBI: National Center for Biotechnology Information
PCA: Principal component analysis
RHG: Rainforest hunter-gatherer
ROHs: Runs of homozygosity
SNP: Single nucleotide polymorphism
VQSR: Variant Quality Score Recalibration
