# Supplementary material for: Genome-wide data from the Bubi of Bioko Island clarifies the Atlantic fringe of the Bantu dispersal
Source: BMC Genomics. 2019 Mar 6;20:179. doi: 10.1186/s12864-019-5529-0 (PMC6404284; doi:10.1186/s12864-019-5529-0)
Supplement: Supplementary file 2 — Supplementary figures. (PDF 1206 kb) [file 12864_2019_5529_MOESM2_ESM.pdf]

Supplementary Figures S1-S12

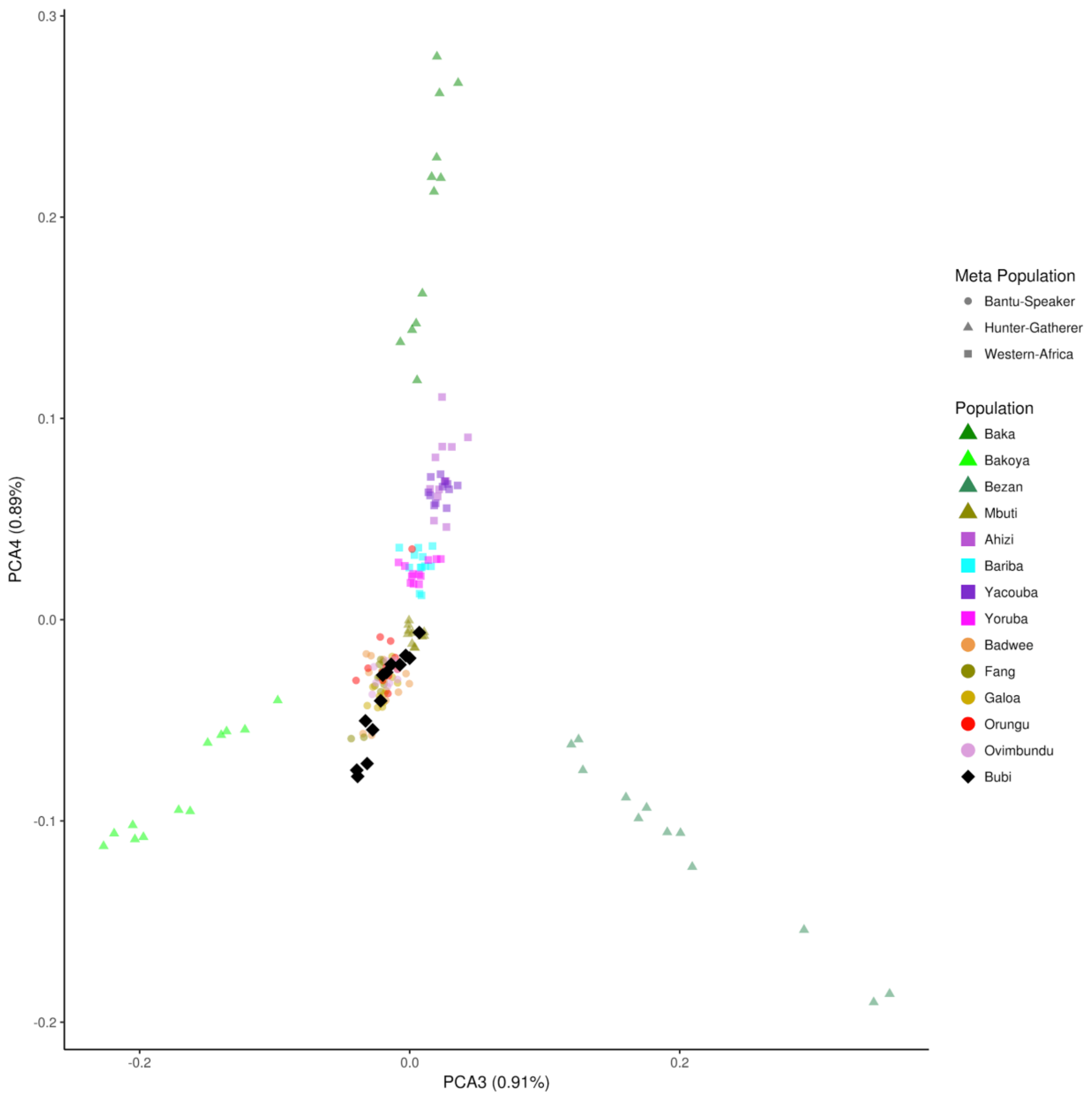

Figure S1: Genotype-based PCA plot obtained with EIGENSOFT smartpca. A dataset of 168 individuals from 14 populations and 581,224 SNPs was used. Only PC3 and PC4 are plotted here. PC1 and PC2 are plotted in Figure 1

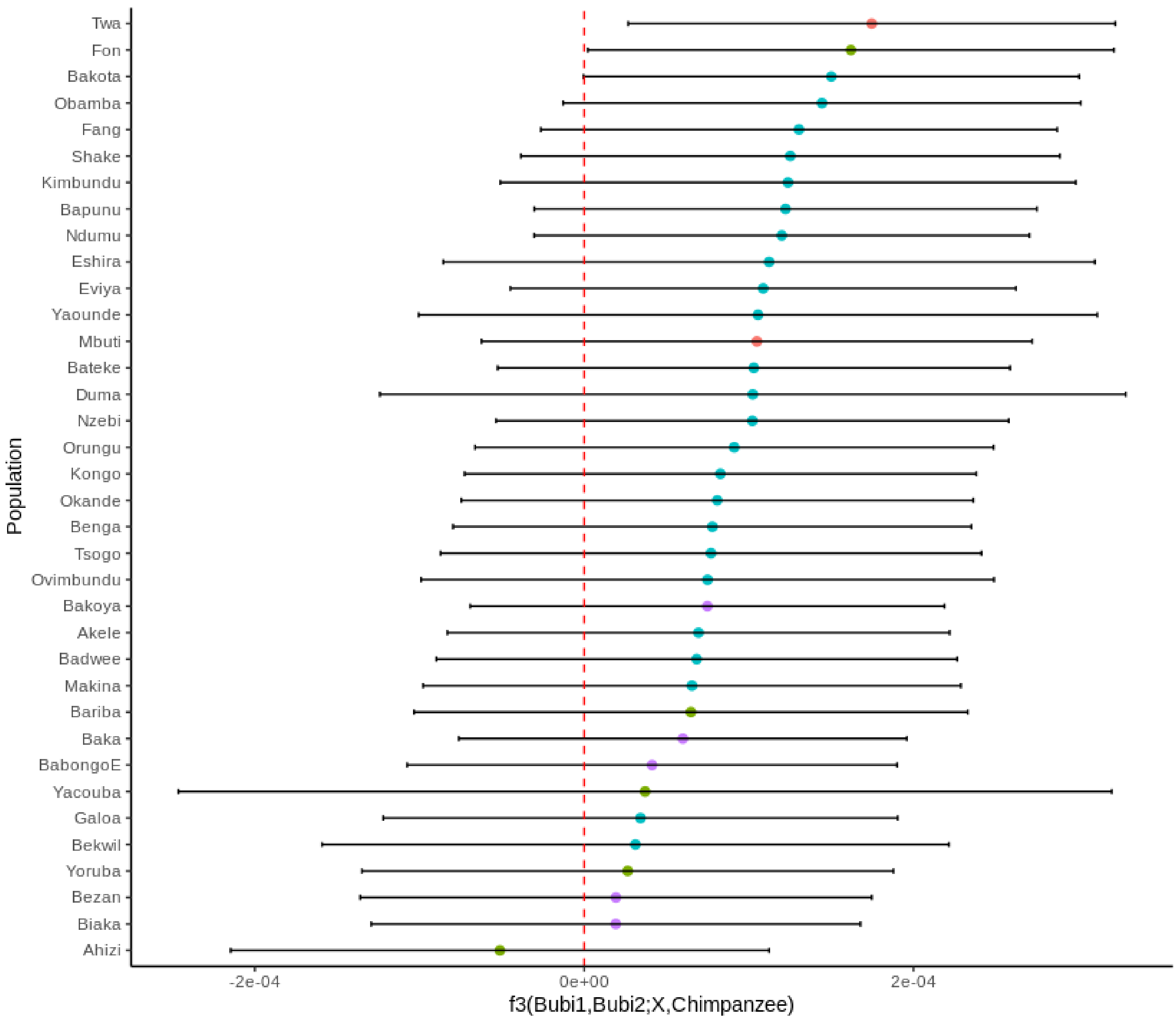

Figure S2: f_4 statistics calculated with popstats software in 36 combinations of populations with the (Bubi1, Bubi2; X, Chimpanzee) population combination. Error bars indicate one standard-deviation. Dot colours indicate Metapopulations: Green for Western African populations, Blue for Western Bantu-Speaking populations, Violet for Western Hunter-Gatherer populations and Brown for Eastern Hunter-Gatherer populations. Bubi people have not been clustered with any metapopulation.

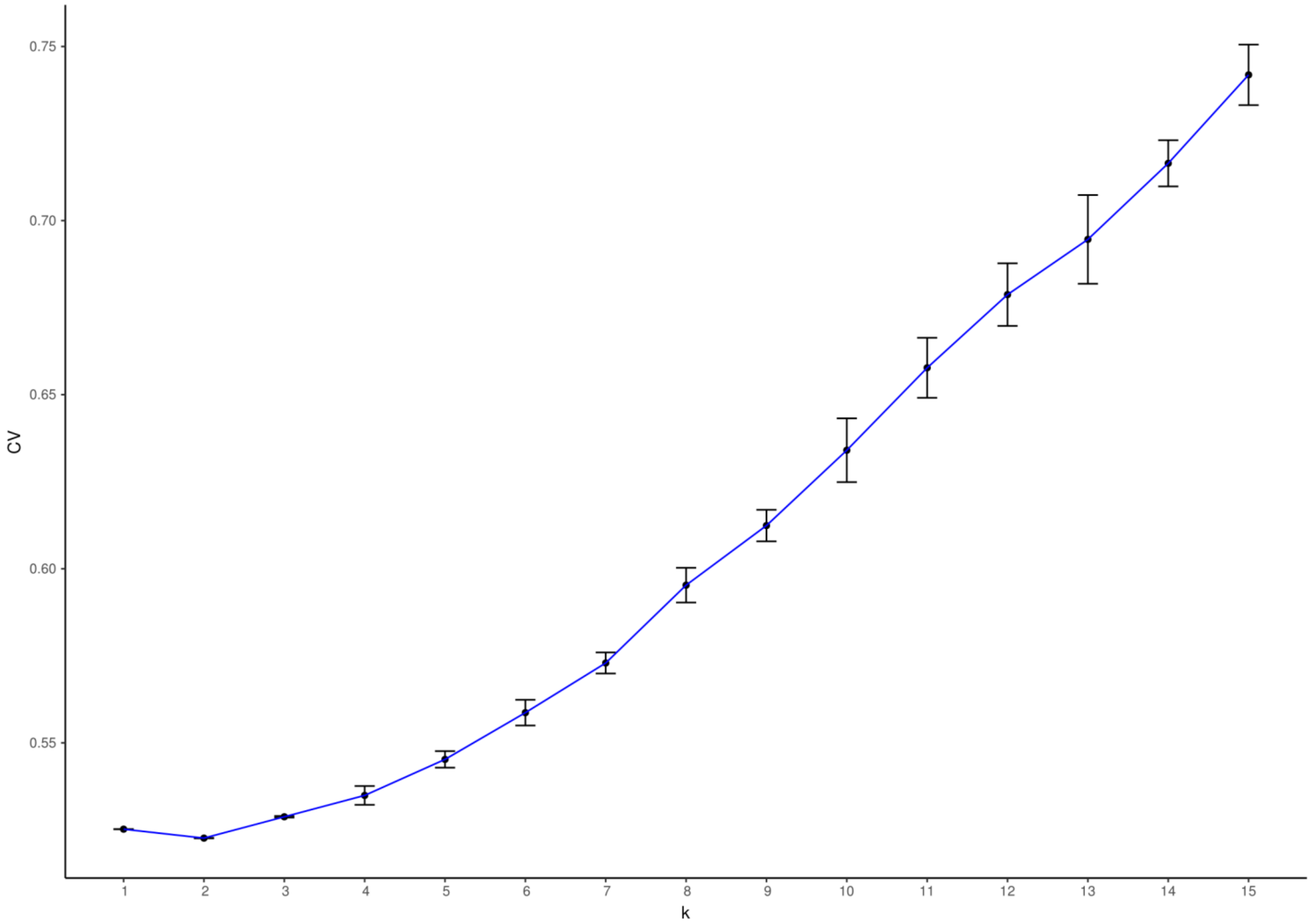

Figure S3: Main values of the Cross-validation errors of the Admixture computations (K:1-15). The black intervals are the deviation of the 10 independent computations performed in each simulation.

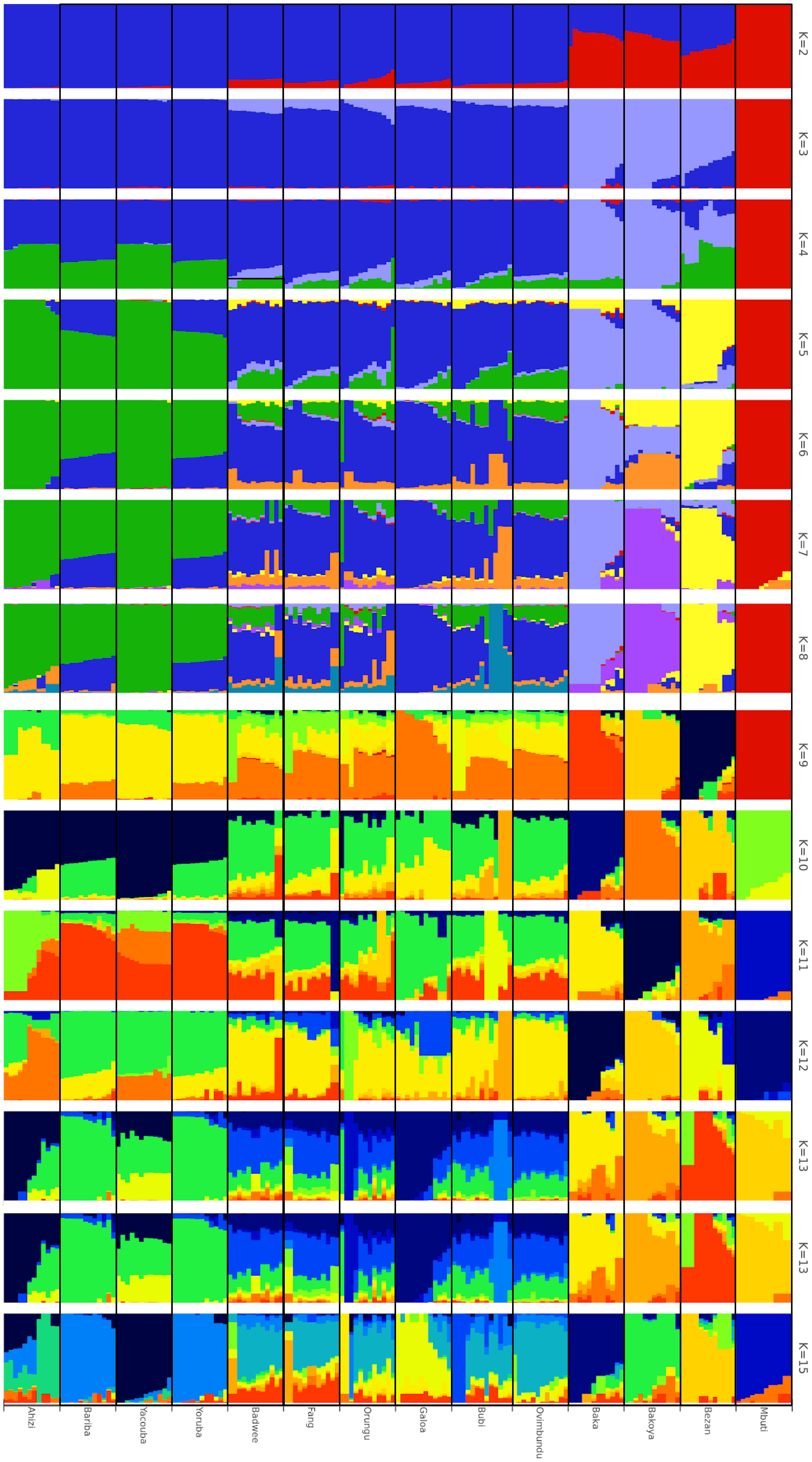

Figure S4: Admixture plots (K= 2-15) created using 456,095 SNPs and 169 individuals from 14 populations. Populations used (Same order as in the plot): Western-African (Ahizi, Bariba, Yacouba, Yoruba), Bantu-Speaker (Badwee, Fang, Orungu, Galoa, Bubi, Ovimbundu), West Hunter-Gatherers (Baka, Bakoya, Bezan) and East Hunter-Gatherers (Mbuti).

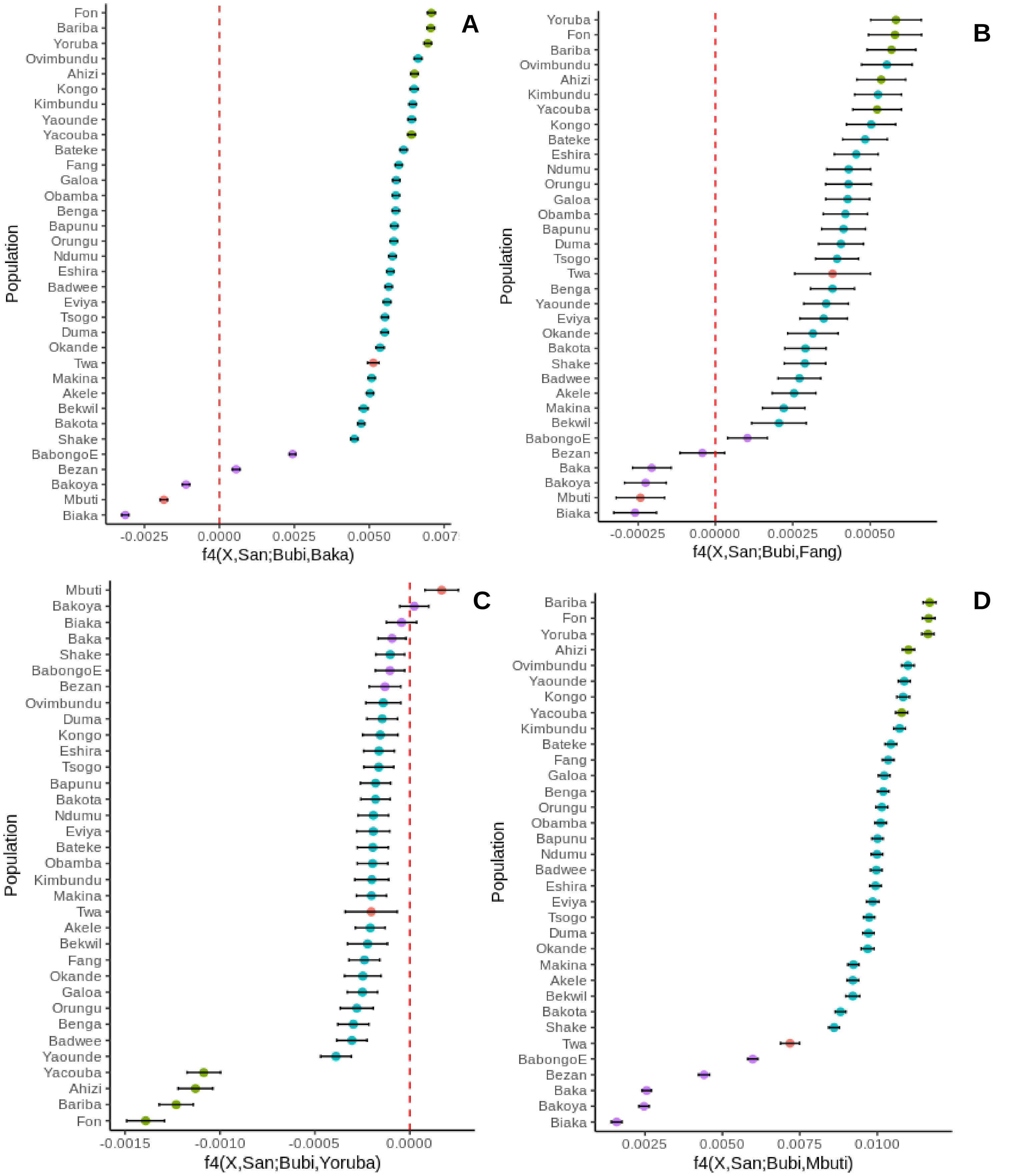

Figure S5: f4 statistics calculated with popstats software in 34 combinations of populations for the combinations: A) f_4(X,San;Bubi,Baka), B) f_4(X,San;Bubi,Fang), C) f_4(X,San;Bubi,Yoruba), D) f_4(X,San;Bubi,Mbuti). Error bars indicate one standard-deviation. Dot colours indicate Metapopulations: Green for Western African populations, Blue for Western Bantu-Speaking populations, Violet for Western Hunter-Gatherer populations and Brown for Eastern Hunter-Gatherer populations. Bubi people have not been clustered with any metapopulation.

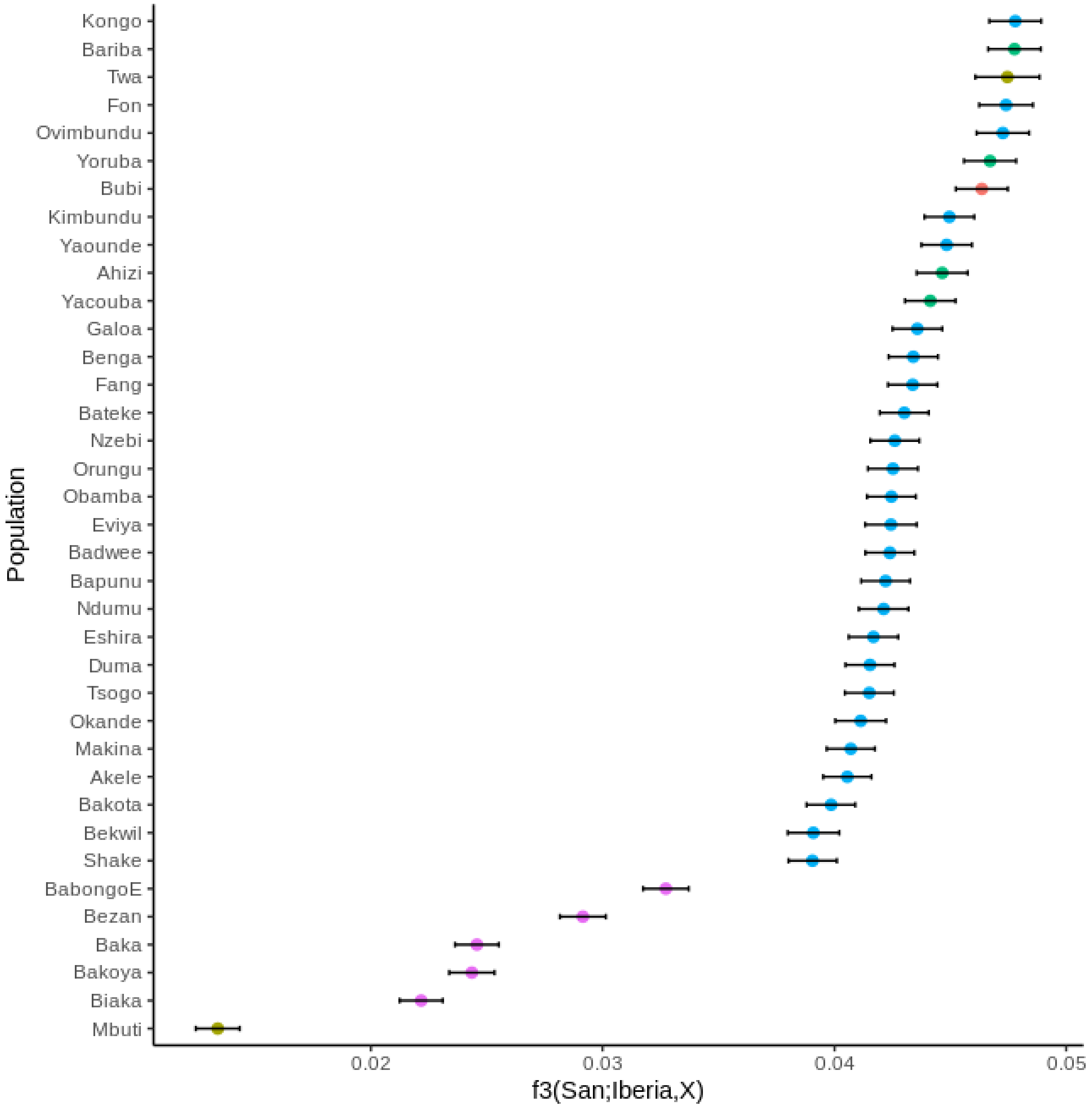

Figure S6: f_3 statistics calculated with popstats software in 36 combinations of populations with the (San; Iberia,X) population combination. Error bars indicate one standard-deviation. Dot colours indicate Metapopulations: Green for Western African populations, Blue for Western Bantu Speaking populations, Pink for Western Hunter-Gatherer populations and Brown for Eastern Hunter-Gatherer populations. Bubi people have not been clustered with any metapopulation.

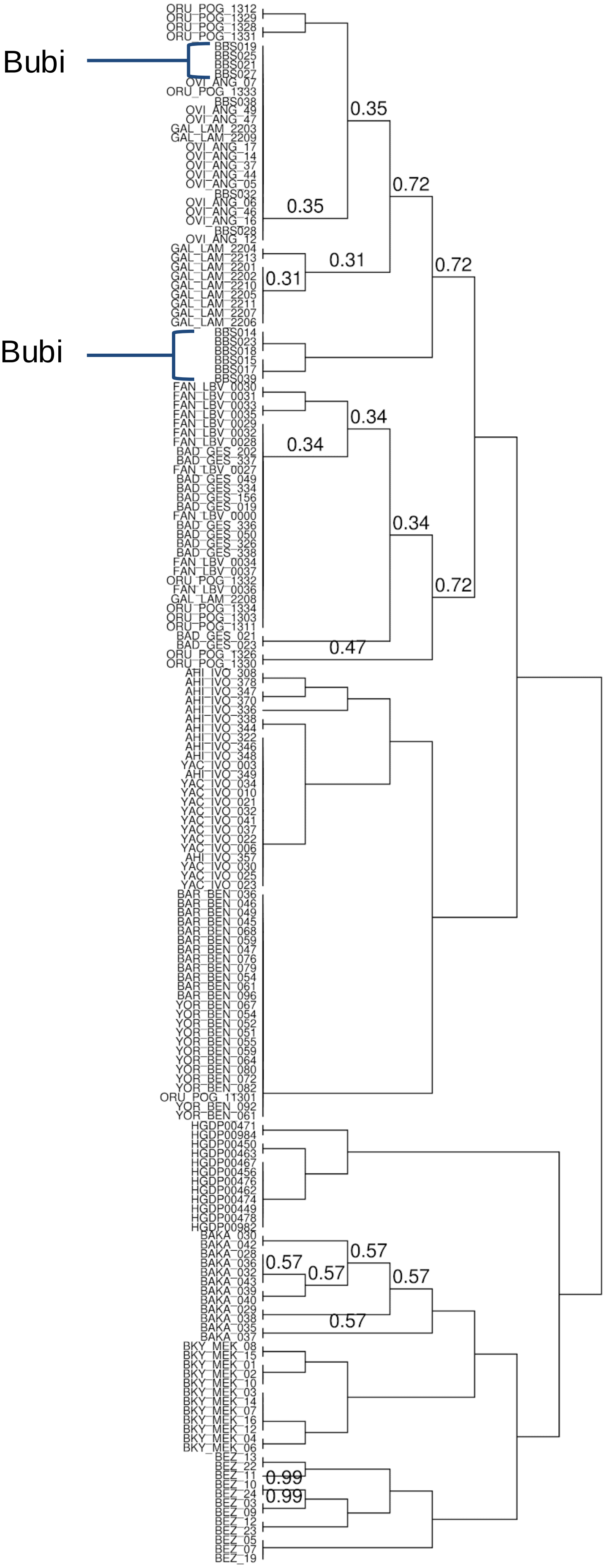

Figure S7: Dendrogram obtained with fineSTRUCTURE. The relation and clustering of individuals is described in Supplementary Table S7. Bubi Individuals have been highlighted.

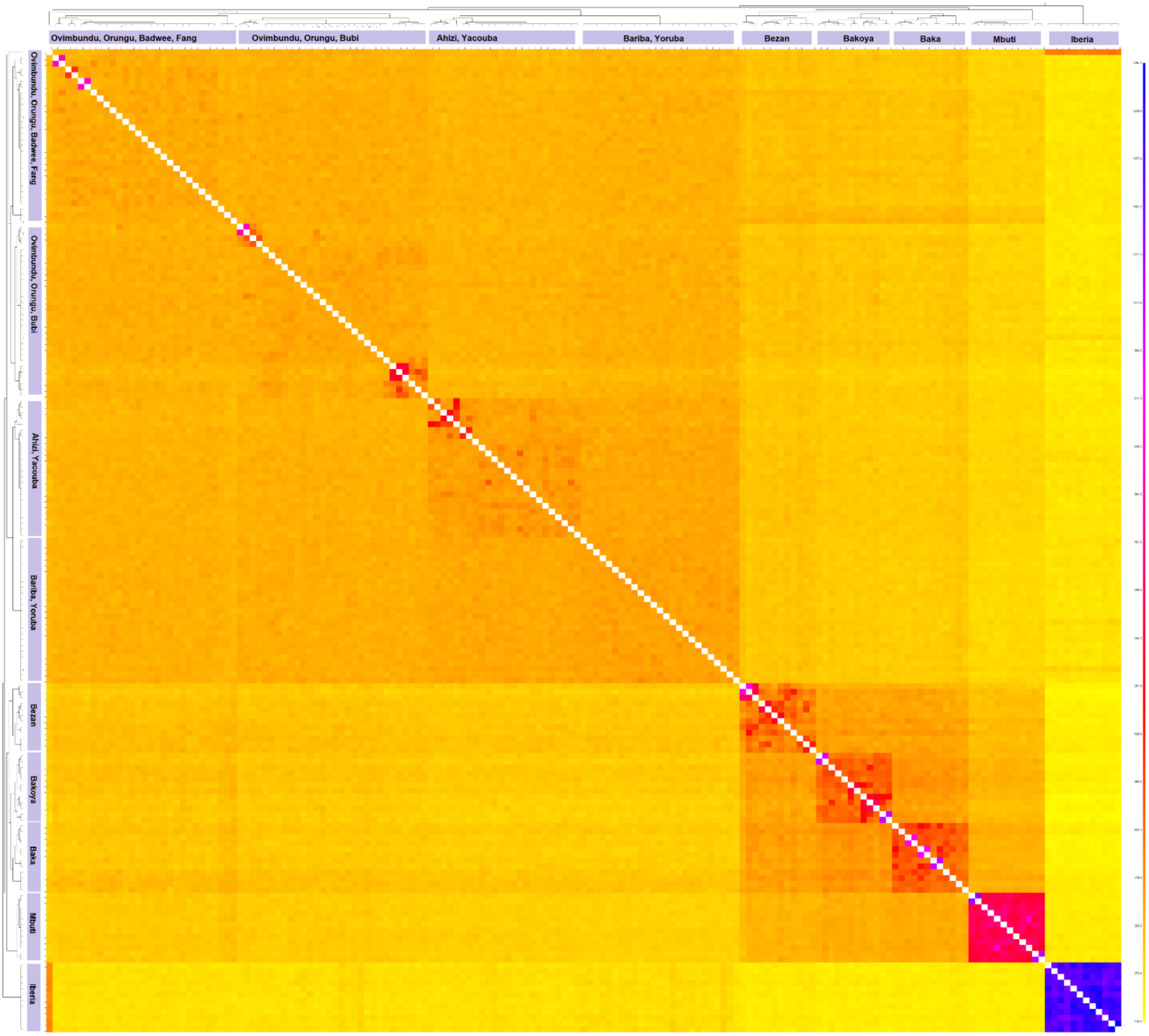

Figure S8: Matrix of shared counts of haplotypes obtained using fineSTRUCTURE. The dataset includes 244,897 phased SNPs. 12 Iberian individuals have been included to the analysis.

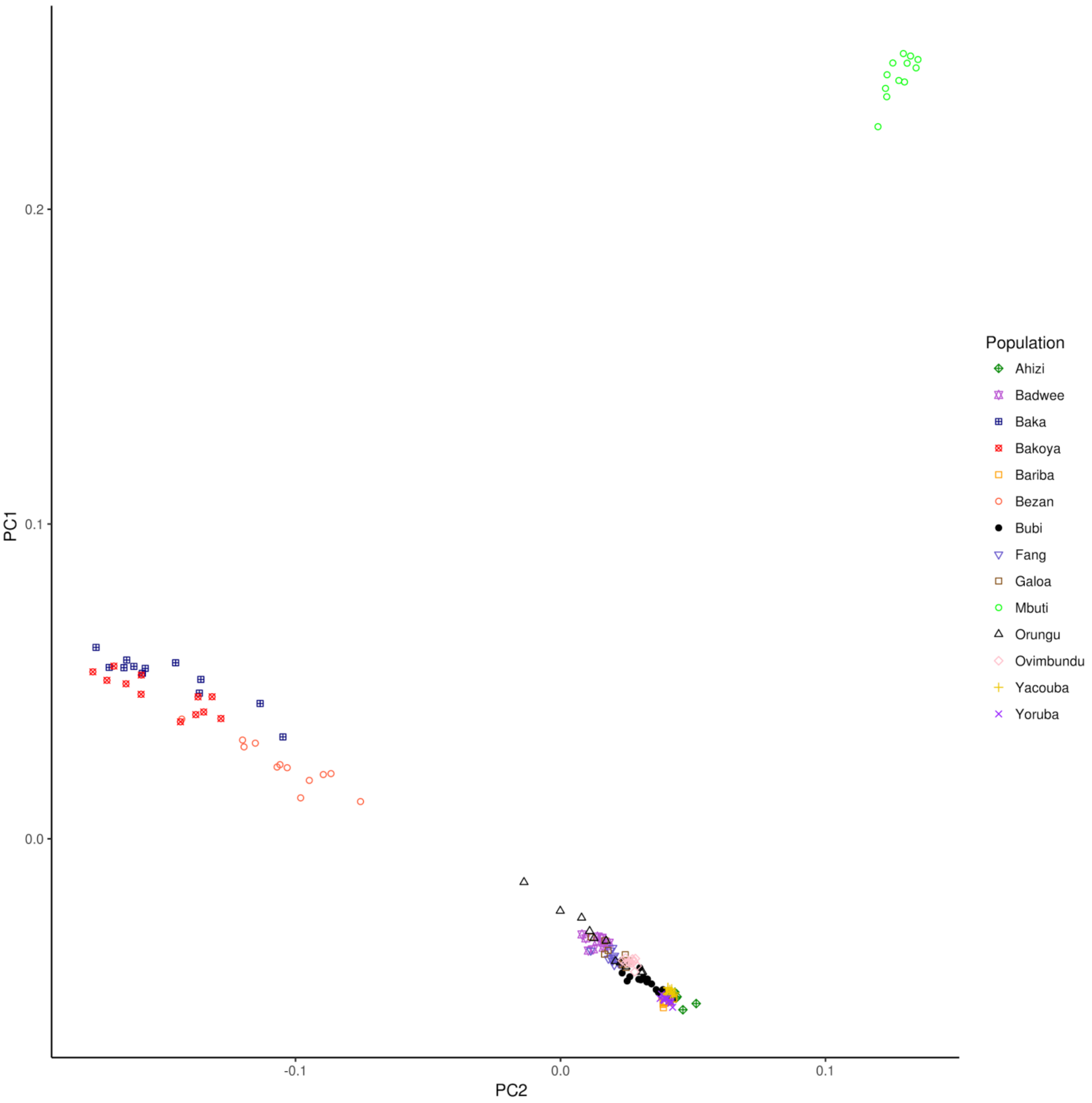

Figure S9: Haplotype based PCA performed with FineSTRUCTURE with 169 individuals and 491,203 SNPs from 14 populations.

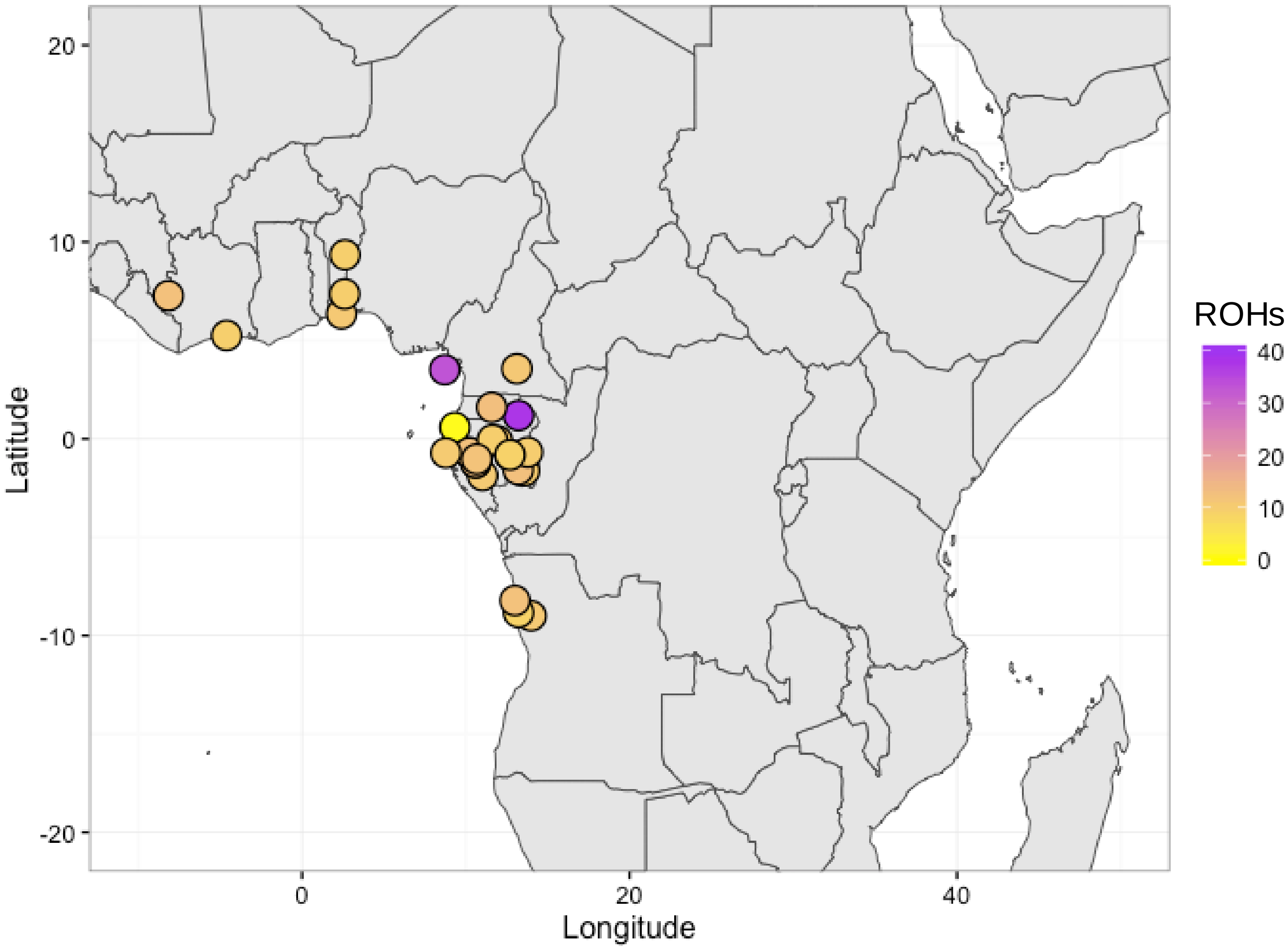

Figure S10: Average counts of individual Runs of Homozygosity (ROHs) per population. Each circle represents one population, only Bantu speaking populations and Bubi are plotted. Bekwil and Bubi populations show the higher averages of ROH per population. The map has been created with R package by one of the coauthors.

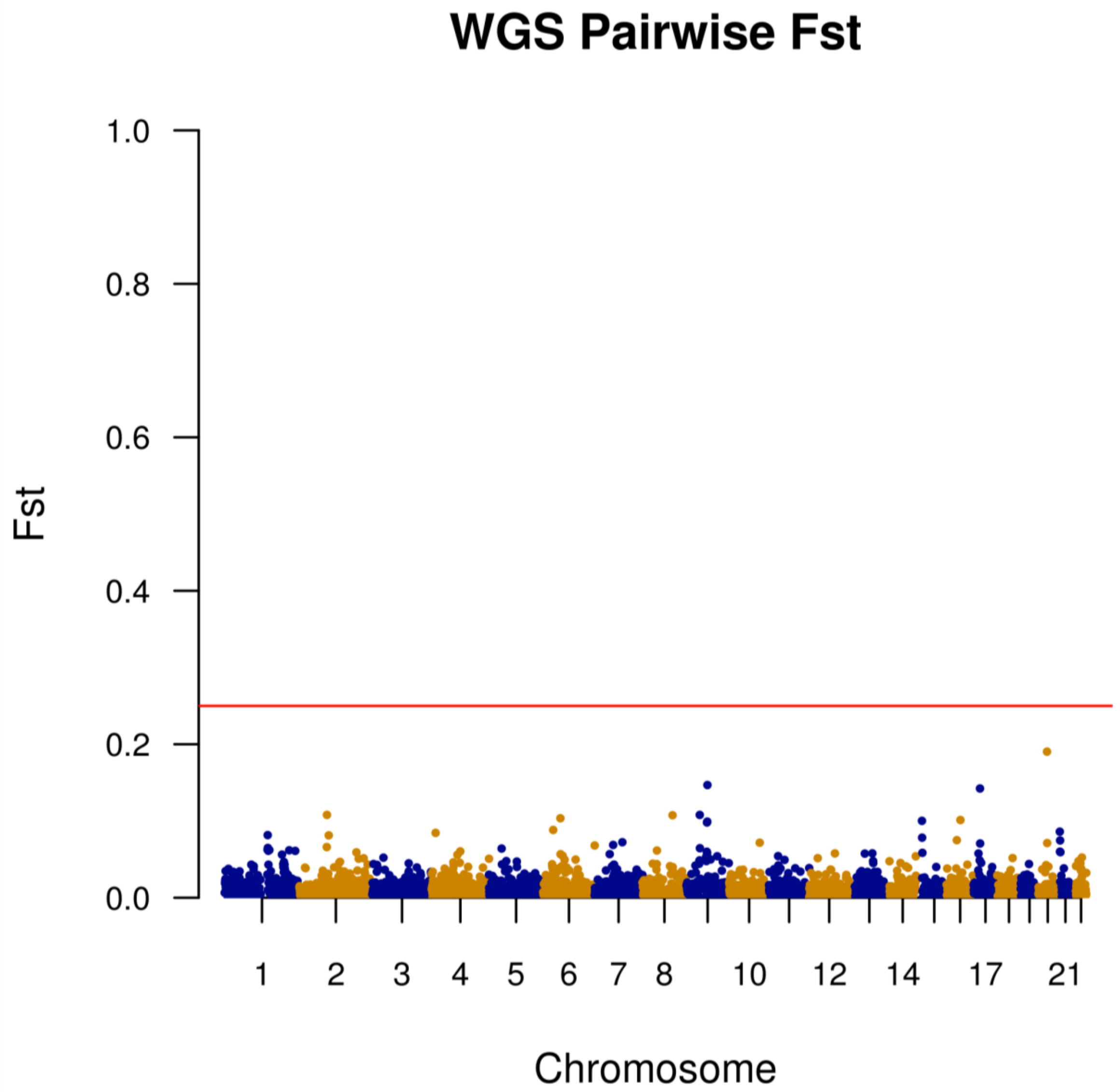

Figure S11: Genome Wide Fst scan for Bubi and Yoruba populations: We computed the pairwise Fst for all the pairs of variable positions of the Genome with MAF > 0.05 and missing genotypes <0.05. The significance threshold has been setted in 0.25.

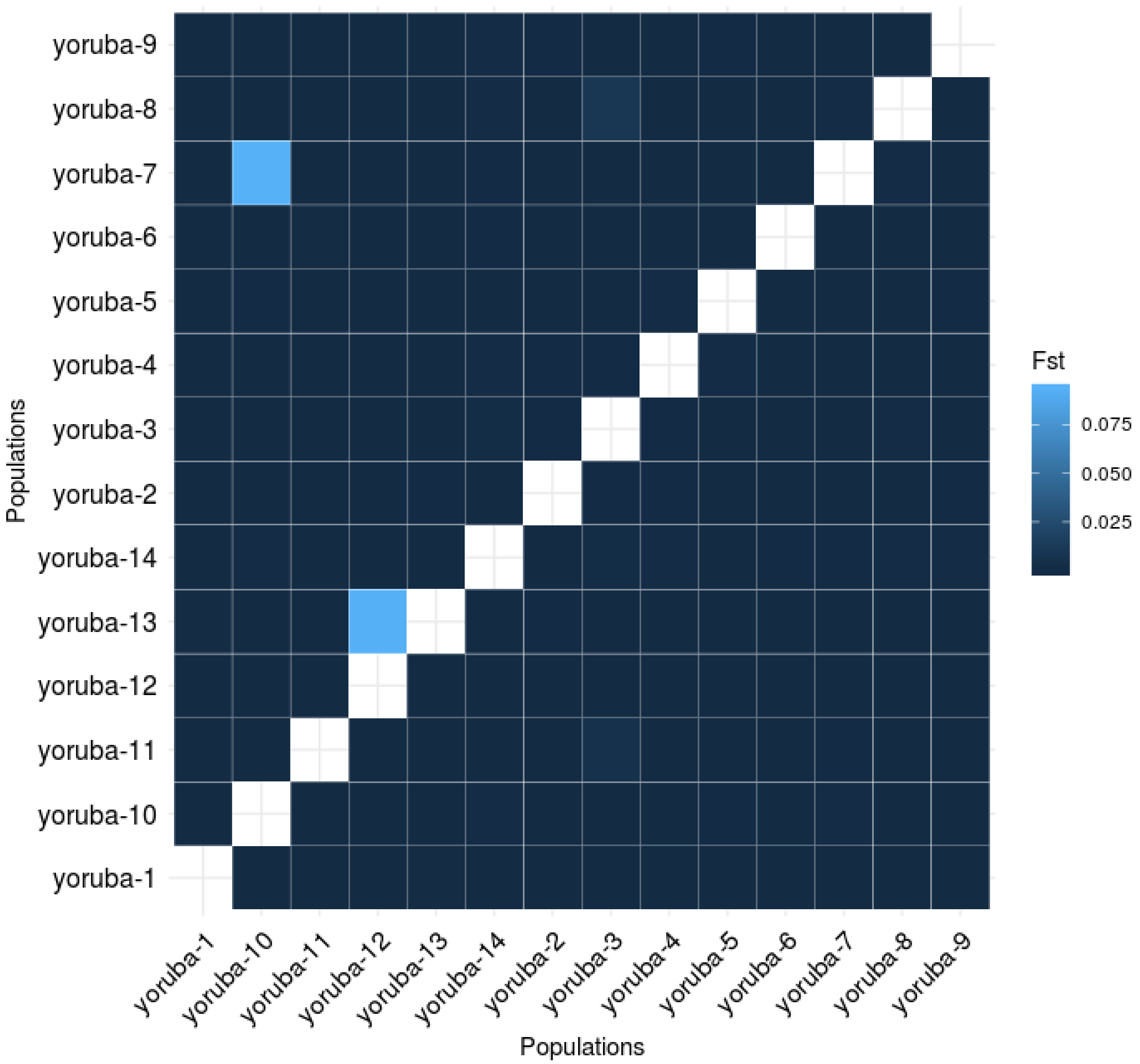

Figure S12: Pairwise genome-wide Fst comparison made with 14 random sub samplings of the 1,000 Genomes Yoruba population in groups of 13-17 individuals. No comparison shows statistically significant values that indicate differences between population sub samplings.
